# Nitrous Oxide

**Published:** 1873-04

**Authors:** G. Q. Colton


### Dental Register.

Vol. XXVII.] APRIL, 1873. No. 4.]

### COMMUNICATIONS.

### Nitrous Oxide.

BY G. Q. COLTON.

The question is still sometimes asked, "Who discovered anæthesia?" To some readers of the DENTAL REGISTER, the following facts may prove interesting.

On the 10th of December, 1844, I gave an "exhibition of the effects of laughing gas, in the city of Hartford, Conn. Among those present, was Dr. Horace Wells, a dentist of Hartford. A young man by the name of Cooley, while under the influence of gas, jumped about, and ran against some wooden settees on the stage. As he sat down next to Dr. Wells, the latter remarked, "You must have hurt yourself;" you run against those benches." Cooley at that moment raised his pantaloons, and was surprised to find his skin bruised and bloody, and declared that he was not aware of having ran against the benches, and felt no pain until the effects of the gas had passed off.

At the close of the exhibition Dr. Wells came to me, and related the experience of Cooley, and asked why a tooth could not be drawn without pain, while the patient was under the influence of this gas. I replied that I did not know, as the thought had never occurred to me. He said he believed it could be done, and he would like to try the experiment, that he would try it on himself first. Accordingly I agreed to take a bag of gas to his office the next day for the purpose. I did so, and Dr. Riggs was called in to perform the operation. I gave the gas to Dr. Wells, and Dr. Riggs extracted an upper molar tooth. On recovering consciousness, Wells slapped his hand on his knee, exclaiming, "*It is the greatest discovery ever made! I did not feel it so much as the prick of a pin!*" This was the first surgical operation ever performed, where an anæsthetic was used to destroy pain—*and the honor of the discovery belongs to Dr. Horace Wells.*

Now the future history of anæsthesia, and especially as connected with sulphuric ether, is somewhat "mixed," but if the reader will note carefully the following facts and dates, he will not have much difficulty in reaching a fair and just conclusion.

After Wells made his first experiment on himself, (December 11th, 1844) I instructed him to make the gas, and then went off on my exhibition tour. About three weeks subsequently, I saw a paragraph going the rounds of the papers, that a Dr. Wells was extracting teeth *without pain* in Boston, while patients were under the influence of laughing gas! After Dr. Wells had tried the gas in Hartford for a few weeks, and satisfied himself of its value as an anæsthetic, he went to Boston to make the discovery known to the scientific world. For this purpose he called upon many physicians, surgeons and dentists, relating what he had done. They all laughed at him, and ridiculed his pretensions. Dr. Warren consented to introduce him to his class in Cambridge College, when, after a brief address, Dr. Wells adminstered the gas to a young man, and extracted a tooth. The young man cried out, though he declared that he experienced no pain. The experiment was pronounced a failure, and Wells was hissed.

Among the dentists upon whom Wells called, was Dr. Morton, his former pupil. Dr. Morton treated him with ridicule, as had all the others. Dr. Wells returned to Hartford, discouraged, and resumed his practice, using the gas, however, in extracting teeth. This he continued till the end of 1845, about one year. We have the testimony of Bishop Brownell and his daughters, as also some forty others of the most respectable citizens of Hartford, that *during this period* Wells extracted teeth for them without pain, using the nitrous oxide gas.

At the close of December, 1845, or early in January, 1846, Wells went to Europe on account of failing health.

In the latter part of September, 1846, Dr. Morton having seen notices of Wells' operations, went to Dr. Jackson, a leading chemist of Boston, to learn how to make laughing gas, as he wished to test the truth or falsity of Wells' pretensions. Jackson said to him, "Why, that gas exhilerates, makes people laugh, dance, etc.; if that will destroy pain, sulphuric ether will do the same. If you wish to try anything, try ether, and avoid the expense of apparatus to make the gas." These were substantially his words. Acting upon this hint, Morton purchased some ether, and, according to all the testimony, *including his own*, Morton's first experiment with it was made on the 30th of September, 1846, almost two years after Wells had used the gas. There is the strongest proof that Wells had tried ether before he went to Europe, but did not like it, preferring the gas.

Morton reported the result of his experiment to Jackson, when they instituted a series of experiments with ether, which, proving successful, they made a joint application for a patent for the discovery of the anæsthetic effects of ether.

This application was signed—*Charles T. Jackson, William T. G. Morton*, and dated "this 27th day of October, A. D. 1846." Before the patent was issued, however, it would seem, that Jackson thought the whole affair might turn to no practical result, and he sold out to Morton his interest in the invention, and wrote to the Commissioner that he had assigned to Morton all his (Jackson's) interest in the patent. The patent was therefore issued to Morton.

When Wells returned to this country, he was astonished to learn that Morton had obtained a patent for the discovery of the anæsthetic powers of ether, and even claimed the honor of *the discovery of anæsthesia!* A discussion on the subject followed between them, in the Boston *Medical Journal.* This discussion, and the mental anxiety in regard to the possibility of losing the honor, which he knew belonged to him, so worked on the sensative nature of Wells, that he became deranged, and died in New York, January 24th, 1848.

At this time no one had used the gas as an anæsthetic, save Wells, and it was some years before the surgical and dental professions recognized and adopted *any* anæsthetic. After the death of Wells, *Morton set up the claim that nitrous oxide was not an anæsthetic*, and that anæsthesia could in no way be produced by it; therefore he discovered anæsthesia. As there was no one at this time who had the inclination, or the necesssary information on the subject, to defend the claims of Wells, Dr. Morton's story was generally believed; and thus many eminent men were led to commit themselves on the subject, in favor of Morton. This state of things continued for twenty years, the nitrous oxide having passed out of the public mind as an anæsthetic, till I *revived* it in 1863. The facts attending this revival are as follows:

Not being a dentist or a surgeon, I had no use for the gas as an anæsthetic. But I remembered the experiment with Wells, and often recounted the above facts in my introductory lectures. On one occasion at New Britain, Conn., during the summer of 1862, after stating the above facts respecting Wells, a lady asked me if I would administer to her the gas, and have a dentist—whose office was in the building—extract some teeth. I consented, and the dentist, whose name escapes me at this moment, extracted teeth not only for this lady, but for two others. This dentist was so delighted with the operation that he insisted upon my instructing him how to make the gas. A year passed, when I returned to New Britain again, in 1863, to give another series of lectures and exhibitions. I learned that this dentist had used the gas during the past year with entire success, giving it, as he stated, to over *six hundred patients?* I told him that he was the first dentist whom I had been able to persuade to try the gas. As I was going to New Haven to lecture, I asked him if he would come there, and we would extract teeth for one week with the gas, as I wished to establish its anæsthetic power. He came, and we commenced in the office of Dr. J. H. Smith, where, with the aid of Dr. Smith, we continued the business for three weeks and two days, in which time we extracted something over three thousand teeth and stumps. This, I thought, was a little better business than lecturing, often to "a miserable account of empty boxes," and determined me to come to New York, and establish an institution devoted exclusively to extracting teeth with the gas. As my name has been for so many years inditified with laughing gas, I called it the "Colton Dental Association."

It will be seen by the above that I claim no honor in the *discovery* of anæsthesia. That honor belongs to Dr. Wells. But I confess to some pride in being the *occasion* of the discovery, and of having given the gas to Dr. Wells for the first operation ever performed with an anæsthetic. If any honor is due me, it is in reviving and establishing the use of the gas, after it had lain dead and forgotten for twenty years.

As to the value of the gas as an anæsthetic, I can present no stronger evidence than the following facts: After I had given the gas for nine months, I commenced, Feb. 4th, 1864, to ask my patients to write their names on a scroll. These names I have numbered regularly from the beginning. The present number on the scroll is *sixty three thousand three hundred and six.* (63,306) In all this number we have never had an accident, or even had to call a carriage to take a patient home. We have sometimes had patients swallow some blood, causing nausea and vomiting; and in rare cases vomiting where no blood was swallowed, owing to some peculiar condition of the stomach, or nervous system. *There has never been a well authenticated case of death from the effects of the gas.*

In a communication in the Register of May last, signed "H," the writer assumes that the death of Mrs. O'Shaughnessy in this city, was caused by the gas. It was proved at the Coroner's inquest that she only *attempted* to inhale the gas three or four times, and finally concluded to have her teeth drawn *without the gas*, which was done. The woman was in a fearful state of nervous excitement, and as much afraid of the gas as the pain. She fainted from fright and shock to the nervous system—an occurrence which has taken place many times before the gas or anæsthesia was thought of. I think Dr. Newbrough, the dentist, made a mistake in not placing the woman flat upon her back as soon as the fainting occurred. At the inquest it was found that the lungs were in a state of congestion, and the verdict of the jury was that death was caused by "asphyxia," which in their opinion had been induced "by gas administered." Now all authorities, including *Prof. Ziegler* and *Prof. Doremus*, lay it down that the quickest remedy for asphyxia is to administer nitrous oxide gas; that instead of producing, it prevents congestion. Prof. Weisse, one of the jurors, stated at a recent meeting, held in the College of Physicians and Surgeons in this city, that the jury used the words "gas administered," and purposely avoided the words "nitrous oxide," as they thought the gas might have been impure, or chloroform might have been put into the bag. In justice to Dr. Newbrough, I should state that there was no evidence that chloroform was used. And I went to his office the next morning, and at his request inhaled some of the gas which was still left in the gasometer, and found it pure gas. It is perfectly clear to my mind that if the woman had taken the gas properly, she would have been alive now. She died because she had her teeth drawn *without* the gas. There would have been no shock to the system, and she could not have fainted. She had not taken as much gas, in all the attempts, as I would give to a child two years old.

A word of advice, in conclusion, to dentists. When *chloroform* is administered, and the patient is carried to the point of stertorous breathing, it is considered a point of danger. But with the gas the point of the stertorous breathing is only the *commencement* of the anæsthetic sleep. Dentists, fearing to give too much, often stop at this point, and by the time they get the instrument on the tooth, the patient is half awake, and, of course, experiences pain.

I would not be understood as saying that there is no danger in the administration of nitrous oxide gas. No doubt injury can be done with it. Caution, judgment, and discretion, are to be exercised. It is very important, also, to have a pure gas. It should always be given from a valve mouth-piece, and not from a bag; though a bag can safely be used for most patients, provided it holds from seven to eight gallons. With the valve mouth-piece, all carbonic acid is avoided.

